# Correspondence

**Published:** 1890-10

**Authors:** N. A. Williams

**Affiliations:** Macon, Fla.


*To the Southern Medical Record :*

In answer to Dr. T. J. Cook's case in the Sept. No., page 415, would say that my diagnosis from his own description is that his patient has what is ordinarily called gall stone colic. I treated a negro woman a few weeks since, quite similar to his, and my treatment was as follows :

℞ Fl. ext. cascara sagrada, ℥ i.

" " mandrake, ℨ ss.

" " ipecac,

" " scillae, aa ℨ ii.

" " nux vomica, ℨ i.

Syrupus simplex, ℥ vi.

S. Tablespoonful every 6 hours till bowels are fairly acted upon, after which it may be necessary to give the medicine less frequently, to keep the whole system in a moderately relaxed condition. In the meantime, I gave quinia in five grain doses every four hours for two or three days, to meet any malarial tendency so often met with in these cases. I also gave iodide potassium in 5-gr. doses well diluted, three times a day, giving directions to arrange the doses as far apart as possible. This is of much importance in the treatment of most diseased conditions of the system. As a febrifuge I used the following, whether fever was present or not :

℞ Bro. pot., ℨ ii.

Spiritus aetheris nitrosi, ℥ i.

Fl. ext. aconite, gtt. xxx.

Aqua pura, ℥ vi.

S. Tablespoonful in a little water every four hours, and after a day or two less frequently, according to symptoms. In a week or ten days I enjoyed the happy surprise of being presented with a number of gall stones the size of a small buck shot, which I now have. This case proceeded to a favorable termination in two or three weeks, and the woman is now enjoying good health.

This woman is the mother of eight or more children, and when she applied for treatment at my office was carrying a sufficient amount of flesh, and the most prominent symptoms were those of jaundice, more or less fever at times, with attacks of sick stomach, poor appetite and occasional attacks of colic. She is about 45 years of age, and of stout build.

N. A. WILLIAMS, M. D., Macon, Fla.

*Sept. 10, 1890.*

